# Localizing Left Atrial Flutter Using the Advisor™ HD Grid Mapping Catheter, Sensor Enabled™

**DOI:** 10.19102/icrm.2021.120109S

**Published:** 2021-01-15

**Authors:** Ahmadreza Karimianpour, Jeffrey R. Winterfield, Joshua E. Payne

**Affiliations:** ^1^Section of Clinical Cardiac Electrophysiology, Division of Cardiology, Department of Medicine, Medical University of South Carolina, Charleston, SC, USA

**Keywords:** Atrial flutter, Advisor HD Grid catheter, high-density mapping, local activation time

High-density electroanatomic mapping can be useful in describing atrial tachycardia mechanisms and in determining successful sites for catheter ablation.

A 75-year-old woman with a history of prior ablation for persistent atrial fibrillation was referred for atrial tachycardia ablation. Based on her history and surface electrocardiogram P-wave morphology, a reentrant left atrial tachycardia was suspected. Isochronal late-activation mapping (ILAM) was performed during coronary sinus pacing using the Advisor™ HD Grid Mapping Catheter, Sensor Enabled™ and EnSite™ NavX™ Precision cardiac mapping system, revealing a deceleration zone along the posterior left atrium with highly fractionated local signals **(Figures 1A and 1D)**. Following the induction of atrial tachycardia, local activation time mapping supported the diagnosis of left atrial reentrant tachycardia **(Figure 1B and Video 1)**. Entrainment was attempted; however, the tachycardia terminated repeatedly with catheter placement. Radiofrequency ablation was performed at this site along with isolation of the posterior wall. The pulmonary veins remained isolated from the previous procedure. The tachycardia was noninducible postablation, with no clinical recurrence at three months of follow-up.

This case highlights the utility of high-density mapping to accurately define tachycardia mechanisms and facilitate successful catheter ablation.

## Figures and Tables

**Figure 1:**
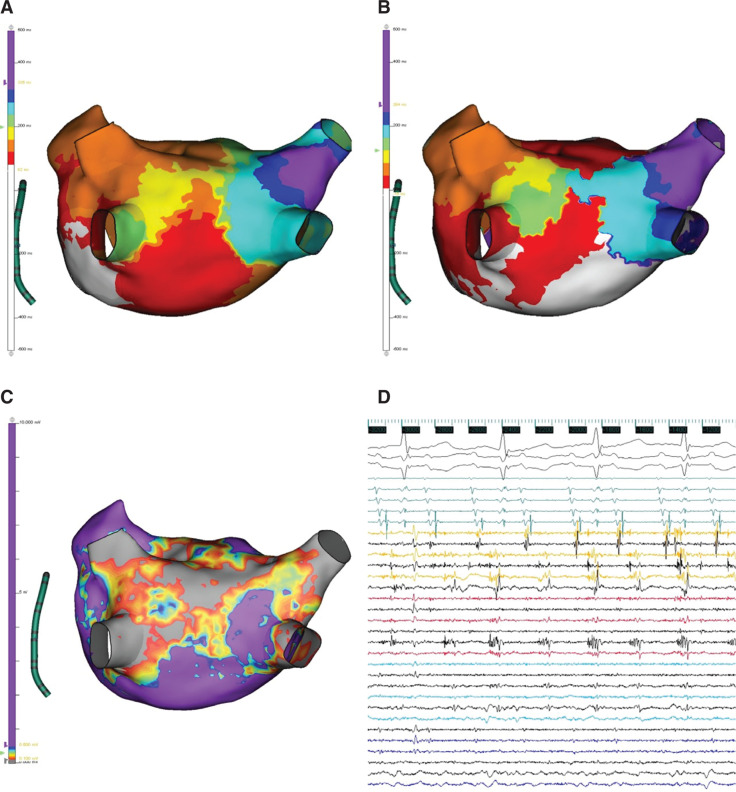
Left atrial electroanatomical maps and intracardiac electrograms. **A:** Isochronal late-activation map (ILAM) obtained during coronary sinus (CS) pacing. **B:** Local activation time during atrial tachycardia. **C:** Voltage map obtained during CS pacing. **D:** Intracardiac electrograms obtained using the Advisor™ HD Grid catheter at the site of termination during tachycardia, showing highly fractionated diastolic potentials.

**Video 1.** Propagation animation of local activation time in the left atrium during atrial tachycardia in a posteroanterior view. A theorized critical isthmus is denoted by (^).

